# Supplementary material for: Parallel encoding of speech in human frontal and temporal lobes
Source: Nat Commun. 2025 Dec 29;17:814. doi: 10.1038/s41467-025-67517-7 (PMC12824166; doi:10.1038/s41467-025-67517-7)
Supplement: Supplementary file 1 — Supplementary Information [file 41467_2025_67517_MOESM1_ESM.pdf]

Supplementary Information

Supplementary Results

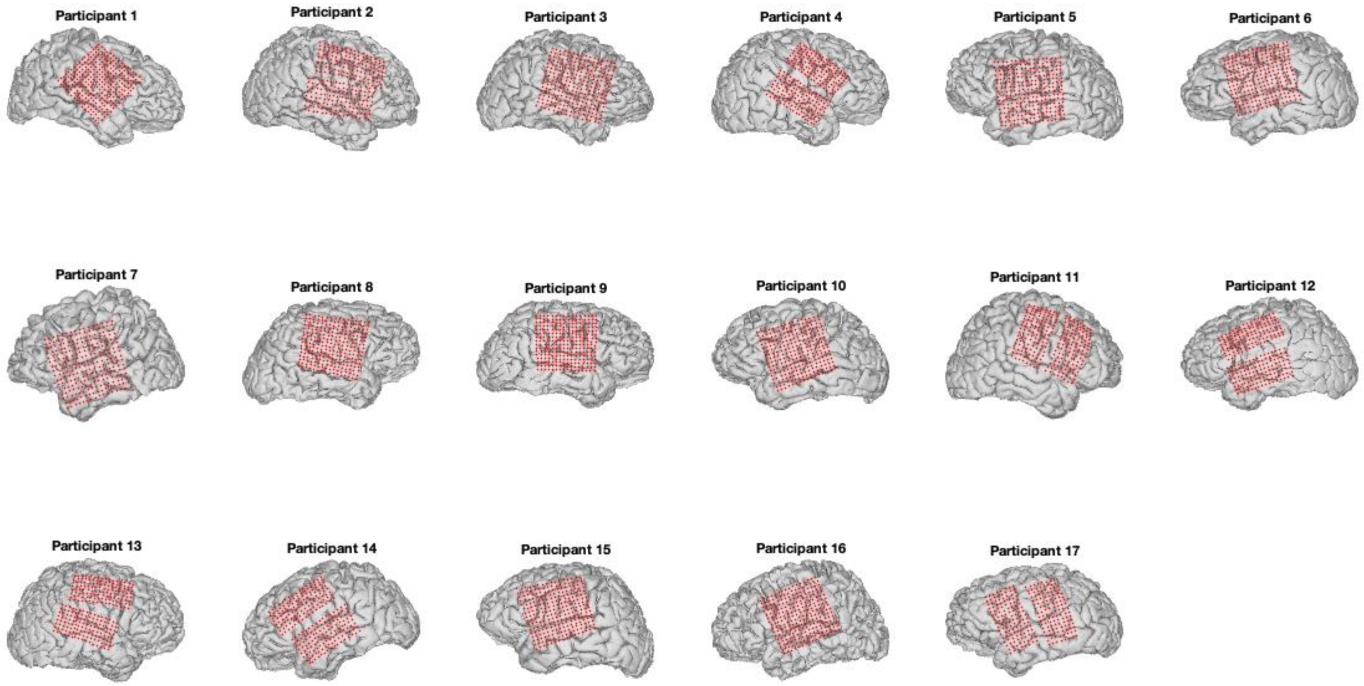

Supplementary Figure 1. Electrode coverage of every participant included in the study. This figure demonstrates the high degree of superior temporal gyrus coverage in each participant which is evidence that synchronous frontal lobe onsets is not secondary to insufficient superior temporal gyrus coverage.

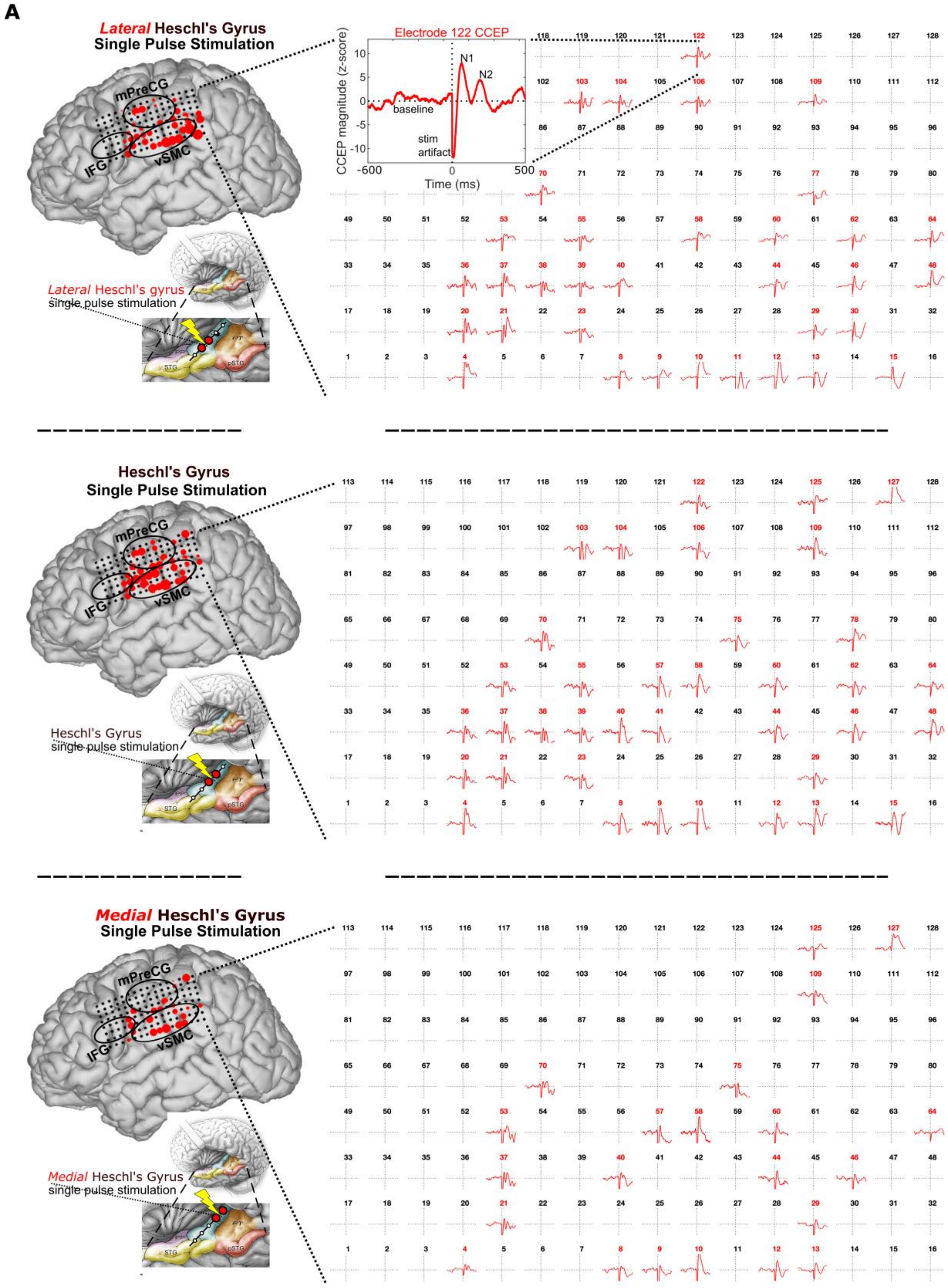
A
Lateral Heschl's Gyrus
Single Pulse Stimulation
Electrode 122 CCEP
CCEP magnitude (z-score)
Time (ms)
N1
N2
baseline
stim
artifact
mPreCG
IFG
vSMC
Lateral Heschl's gyrus
single pulse stimulation
Heschl's Gyrus
Single Pulse Stimulation
Heschl's Gyrus
single pulse stimulation
Medial Heschl's Gyrus
Single Pulse Stimulation
Medial Heschl's Gyrus
single pulse stimulation
PP
STG
PT
pSTG

Supplementary Figure 2. **A.** Single pulse stimulation was performed along primary auditory cortex in lateral Heschl's gyrus (top panel), mid-Heschl's gyrus (middle panel), and the medial aspect of Heschl's gyrus (bottom panel). Concurrent recordings were made in the frontal and parietal lobes with a high-density 128-channel grid. Each cortico-cortical evoked potential (CCEP) is shown on the right. Only sites with significant CCEPs are shown ($p < 0.0001$, Wilcoxon rank sum test, one-sided, $n = 30$ stimulated CCEPs at each site). Significance was defined by comparing the absolute value of the mean baseline potential (-300 to -50ms) to the mean of the absolute value after stimulation artifact (+25ms to +275ms). The size of the red circles on the brain plots (●) is proportional mean of the absolute valued CCEP[1]. As shown a sparse distribution of sites have significant CCEPs in the frontal lobe, from Heschl's gyrus stimulation, and these sites tend to cluster in the previously defined frontal lobe areas: middle precentral gyrus (mPreCG), ventral sensorimotor cortex (vSMC), and inferior frontal gyrus (IFG). This data is consistent with parallel projections from Heschl's gyrus (primary auditory cortex) to these frontal lobe areas. This figure contains brain illustrations from Kenneth Probst, reproduced with permission, licensed under a Creative Commons Attribution 4.0 https://creativecommons.org/licenses/by/4.0/deed.en

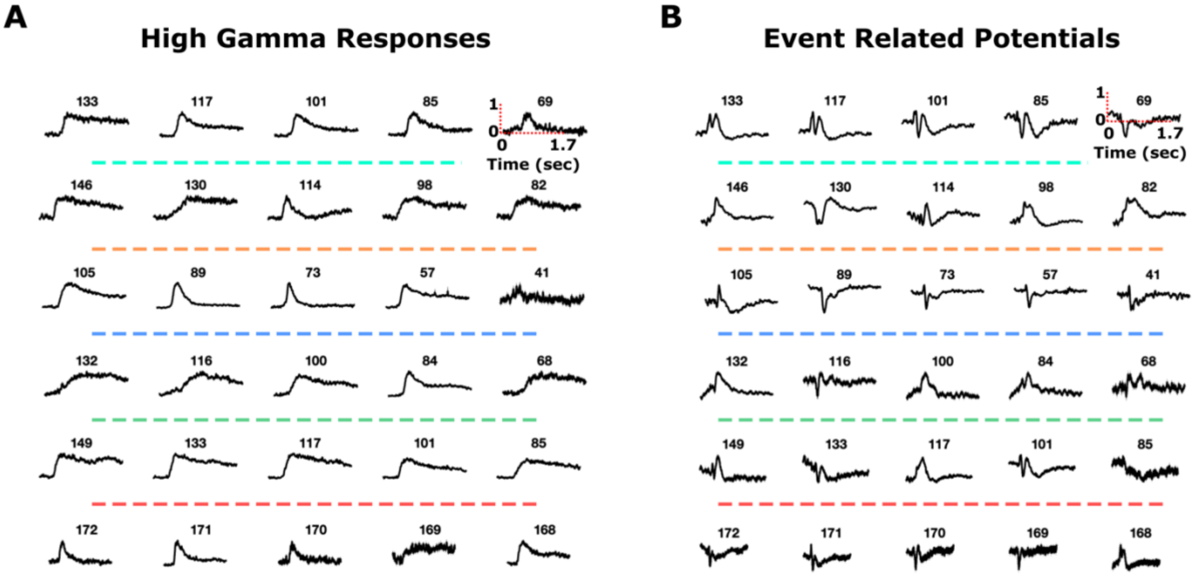

Supplementary Figure 3. Contrast between high-gamma responses **(A)** compared to high waveform morphology of ERPs **(B)** calculated at the same electrodes. Each row is from a different participant. Each response represents the average to 400 sentences ($n = 400$).

Because the high-gamma band reflects local neuronal spiking[2–5], it has been used extensively in prior work, including to investigate onset latencies in the human auditory system[6]. We use high-gamma activity in this manuscript to compute the onset of local neuronal spiking under each electrode and decode the information in those responses to make claims about when neurons in these areas respond to acoustic input.

While raw voltage evoked response potentials (ERPs) contain important information about neural activity and were considered in the analysis, they have only an indirect relationship with local neuronal firing rates[3–5]. ERPs result from pooled activity over larger volumes of cortex due to increased volume conduction of lower frequency bands [7–9] and are poorly correlated with local neuron firing rates under electrodes where the ERP was recorded[5].

In addition, due to the above points, ERPs often have a highly variable and multiphasic profile which makes quantification of onset latencies challenging. In supplementary figure 3 we show examples of the high-gamma and raw voltage evoked responses to speech from several participants. As is apparent on the right, the multiphasic raw voltage response makes computing and interpreting onset latencies challenging. Although the raw voltage has an advantage of not being affected by smearing associated with filtering, we have addressed this concern in our use of Morelet Wavelets to compute the high-gamma signal (see Methods in the main manuscript).

Supplementary Methods

Cortico-cortical evoked potentials

For each Heschl's gyrus stimulation site, a constant-current square wave pulse (100 μS) was applied to two adjacent electrodes[10]. Stimulation intensity was titrated up to a target current level of 10mA without after-discharges. Continuous real-time ECoG monitoring was used to detect after-discharges or seizures during single-pulse stimulation. No after-dischargers or seizures were triggered. Cortico-cortical evoked potentials (CCEPs) were recorded using a 1–1000 Hz bandpass filter and a sampling rate of 3052 Hz. Concurrent recordings were made in the frontal and parietal lobes with a high-density 128-channel grid. Evoked 1Hz single-pulse stimulation responses were recorded for 30 seconds at each site (30 responses each). Subjects remained at rest or engaged in normal activities without specific tasks during recording.

References

1. Dionisio, S. *et al.* Connectivity of the human insula: A cortico-cortical evoked potential (CCEP) study. *Cortex* **120**, 419–442 (2019).
2. Manning, J. R., Jacobs, J., Fried, I. & Kahana, M. J. Broadband shifts in local field potential power spectra are correlated with single-neuron spiking in humans. *J. Neurosci.* **29**, 13613–13620 (2009).
3. Ray, S. & Maunsell, J. H. R. Different origins of gamma rhythm and high-gamma activity in macaque visual cortex. *PLoS Biol.* **9**, e1000610 (2011).
4. Steinschneider, M., Fishman, Y. I. & Arezzo, J. C. Spectrotemporal analysis of evoked and induced electroencephalographic responses in primary auditory cortex (A1) of the

awake monkey. *Cereb. Cortex* **18**, 610–25 (2008).

5. Leonard, M. K. *et al.* Large-scale single-neuron speech sound encoding across the depth of human cortex. *Nature* **626**, 593–602 (2023).
6. Nourski, K. V. *et al.* Functional organization of human auditory cortex: Investigation of response latencies through direct recordings. *Neuroimage* **101**, 598–609 (2014).
7. Kajikawa, Y. & Schroeder, C. E. How local is the local field potential? *Neuron* **72**, 847–858 (2011).
8. Liu, J. & Newsome, W. T. Local field potential in cortical area MT: Stimulus tuning and behavioral correlations. *J. Neurosci.* **26**, 7779–7790 (2006).
9. Chang, E. F. Towards large-scale, human-based, mesoscopic neurotechnologies. *Neuron* **86**, 68–78 (2015).
10. Matsumoto, R. *et al.* Functional connectivity in the human language system: A cortico-cortical evoked potential study. *Brain* **127**, 2316–2330 (2004).
